# Effects of osteopathic manipulative treatment associated with pain education and clinical hypnosis in individuals with chronic low back pain: study protocol for a randomized sham-controlled clinical trial

**DOI:** 10.1186/s13063-022-07040-y

**Published:** 2022-12-30

**Authors:** Guilherme Luis Santana Luchesi, Anne Kastelianne França da Silva, Otávio Henrique Borges Amaral, Vanessa Cristina Godoi de Paula, Fabrício José Jassi

**Affiliations:** 1 Postgraduate Program in Human Movement Sciences at the Health Sciences Center of the State University of the North of Paraná, Alameda Padre Magno, n° 841 - Nova Jacarezinho, Jacarezinho, Paraná 86400-000 Brazil; 2 Docusse Institute of Osteopathy and Manual Therapy (IDOT), Presidente Prudente, São Paulo, Brazil; 3 Post-doctoral Program of São Paulo State University (UNESP), Faculty of Science and Technology, Presidente Prudente, São Paulo, Brazil

**Keywords:** Manipulation, Osteopathic, Patient Education as Topic, Hypnosis, Low back pain

## Abstract

**Background:**

Patients with chronic low back pain (CLBP) suffer with functional, social, and psychological aspects. There is a growing number of studies with multimodal approaches in the management of these patients, combining physical and behavioral therapies such as osteopathic manipulative treatment, associating pain education and clinical hypnosis. The aim of the present study will be to evaluate the effects of osteopathic manipulative treatment (OMT) associated with pain neuroscience education (PNE) and clinical hypnosis (CH) on pain and disability in participants with CLBP compared to PNE, CH, and sham therapy.

**Methods:**

A randomized controlled clinical trial will be conducted in participants aged 20–60 years with CLBP who will be divided into two groups. Group 1 will receive PNE and CH associated with OMT, and G2 will receive PNE, CH, and sham therapy. In both groups, 4 interventions of a maximum of 50 min and with an interval of 7 days will be performed. As primary outcomes, pain (numerical pain scale), pressure pain threshold (pressure algometer), and disability (Oswestry Disability Questionnaire) will be evaluated and, as a secondary outcome, global impression of improvement (Percent of Improvement Scale), central sensitization (Central Sensitization Questionnaire), biopsychosocial aspects (Start Beck Toll Questionnaire), and behavior of the autonomic nervous system (heart rate variability) will be assessed. Participants will be evaluated in the pre-intervention moments, immediately after the end of the protocol and 4 weeks after the procedures. Randomization will be created through a simple randomized sequence and the evaluator will be blinded to the allocation of intervention groups.

**Discussion:**

The guidelines have been encouraging multimodal, biopsychosocial approaches for patients with CLBP; in this sense, the results of this study can help clinicians and researchers in the implementation of a model of treatment strategy for these patients. In addition, patients may benefit from approaches with minimal risk of deleterious effects and low cost. In addition, it will enable the addition of relevant elements to the literature, with approaches that interact and do not segment the body and brain of patients with CLBP, allowing new studies in this scenario.

**Trials registration:**

Date: September 4, 2021/Number: NCT05042115.

## Background

Low back pain (LBP) stands out as the main cause of disability and for the decrease in world productivity within 354 medical conditions evaluated [1]. The chronification of these cases, in addition to the socioeconomic impacts, is characterized by a complex interaction of biological, social, and psychological factors [1, 2]. In Brazil, the prevalence of CLBP is increasing [3], with findings ranging from 10 to 50% in different populations [4], which can result in a higher prevalence of disability and functional limitations, especially in the economically active population, contributing to negative socioeconomic impacts. Carregaro et al. [5] highlighted that in countries such as Brazil, between 2012 and 2016, about US$ 2.2 billion was spent on CLBP, with 79% of this amount spent on productivity losses, in addition the authors point out that in the same period more than 880,000 imaging exams were performed, and that these individuals missed work 59 million days in that period. These data reinforce the need for improvements in health services and policies to deal with this dysfunction, as well as strategies to reduce the widespread use of diagnostic imaging.

Treatment options that address only single etiologic conditions have been shown to be ineffective. Therefore, multimodal and multidisciplinary approaches to the management of chronic low back pain (CLBP) have been encouraged [6].

Guidelines point out as main treatments for LBP approaches with exercises, education, and manual therapy. Strong evidence recommends aerobic and strength exercise modalities for CLBP, as they promote the resumption of physical capacities and gradual exposure of patients to movements. On the other hand, the education of individuals with CLBP aims to help understand the causes and factors that influence pain intensity, in addition to promoting knowledge about available treatment strategies. Finally, manual therapy can also compose the treatment of this individual, since it allows improvement in movement and spinal stiffness in patients with CLBP [7]. Thus, there is a growing number of clinical trials investigating multimodal approaches in patients with chronic pain.

Osteopathic manipulative treatment (OMT) is a hand-on part of osteopathic approach and a modality used in the treatment of LBP. Dal Farra et al., in a meta-analysis, presented strong evidence on pain reduction and improvement in function in LBP patients undergoing OMT [8]. The results presented by the systematic review by Louw et al. showed that, combining movement therapies, such as manual therapies, with education processes, it potentiated the effects on pain, function, disability, and psychosocial factors and also enabled improvement in movements and decreased use of health services in patients with chronic musculoskeletal pain [9].

Buttler and Moseley developed a method called pain neuroscience education (PNE). Addressing concepts of neurophysiology, neurobiology, representation, and meaning of pain, PNE aims to desensitize the neural system, contrary to the traditional anatomical and biomedical model [10]. In association with educational strategies, Rizzo et al. showed that clinical hypnosis (CH) enhances the results of education processes in patients with CLBP in the short and medium terms [11]. Thompson et al. in a meta-analysis totaling 3632 patients showed the effectiveness of CH as a pain re-education technique, producing moderate to great analgesia for all pain outcomes [12].

In view of the absence of standards in isolated allopathic or complementary treatments in the effectiveness of the treatment of CLBP, the growing number of researches in these areas of activity, and the absence of high-quality clinical trials that combine the aforementioned therapies in the management of this condition, our research is justified. This study aims to know whether OMT associated with PNE and CH is effective in the pain and disability of patients with CLBP in the short and medium terms or if the PNE and CH are sufficient in the management of these patients. We hypothesize that the combination of a physical approach such as OMT with PNE and CH can bring more benefits to this population, improving pain levels and reducing disabilities, in the short and medium terms.

## Objectives

### Primary objective

The primary objective of the present study will be to evaluate the effects of PNE and CH associated with OMT on pain and disability in patients with CLBP compared to PNE and CH associated with sham therapy.

### Secondary objective

The secondary objectives will be to evaluate the effects of PNE and CH associated with OMT on patient's global impression of improvement, central sensitization, biopsychosocial factors, and the modulation of the autonomic nervous system.

## Methods

### Study design and participants

This protocol is a randomized, parallel-group, evaluator-blind, sham-controlled clinical trial, with an allocation ratio of 1:1, prospectively registered at ClinicalTrials.gov (NCT NCT05042115) on September 4, 2021, and follows SPIRIT 2013 and TIDiER-placebo recommendations [13, 14] for clinical trials.

Individuals diagnosed with nonspecific CLBP for at least 3 months will be included, with a mean numerical pain scale (NPE) score of at least 3 points and aged between 20 and 60 years [11]. Individuals undergoing concomitant physiotherapeutic treatment, individuals with medical contraindication to exercise, smokers/alcoholics, severe vertebral or neural pathologies, previous spine surgeries, cardiorespiratory disease, CLBP as a secondary complaint, pregnancy, hearing problems, or illiteracy and those who present an increase in symptoms in the stages of the study will be excluded.

On the day of the initial assessment (M0), all eligible participants will be informed about the procedures and objectives of the study and, after agreeing, they will sign an informed consent form (ICF), being able to voluntarily interrupt their participation in the study at any experiment stage. All study procedures were approved by the Research Ethics Committee at State University of the North of Paraná - UENP (CAEE: 43063621.3.0000.8123) and followed the Declaration of Helsinki.

The procedures will be carried out in reserved rooms of the same clinic in order to avoid any type of exposure or possible embarrassment to the participants. In the event of the possibility or presence of any type of real or potential embarrassment perceived by the participants or researchers, the intervention will be stopped immediately. All procedures will be conducted by the researcher (F.J.J.) with experience of more than 7 years in the proposed procedures, and the evaluation will be conducted by one professional of clinical physiotherapy at the State University of the North of Paraná, previously trained in outcomes measurement. To characterize the participants, the following data will be collected: gender, age, duration of pain (months), weight, height, physical activeness, and use of medication.

### Interventions

**Fig. 1:**
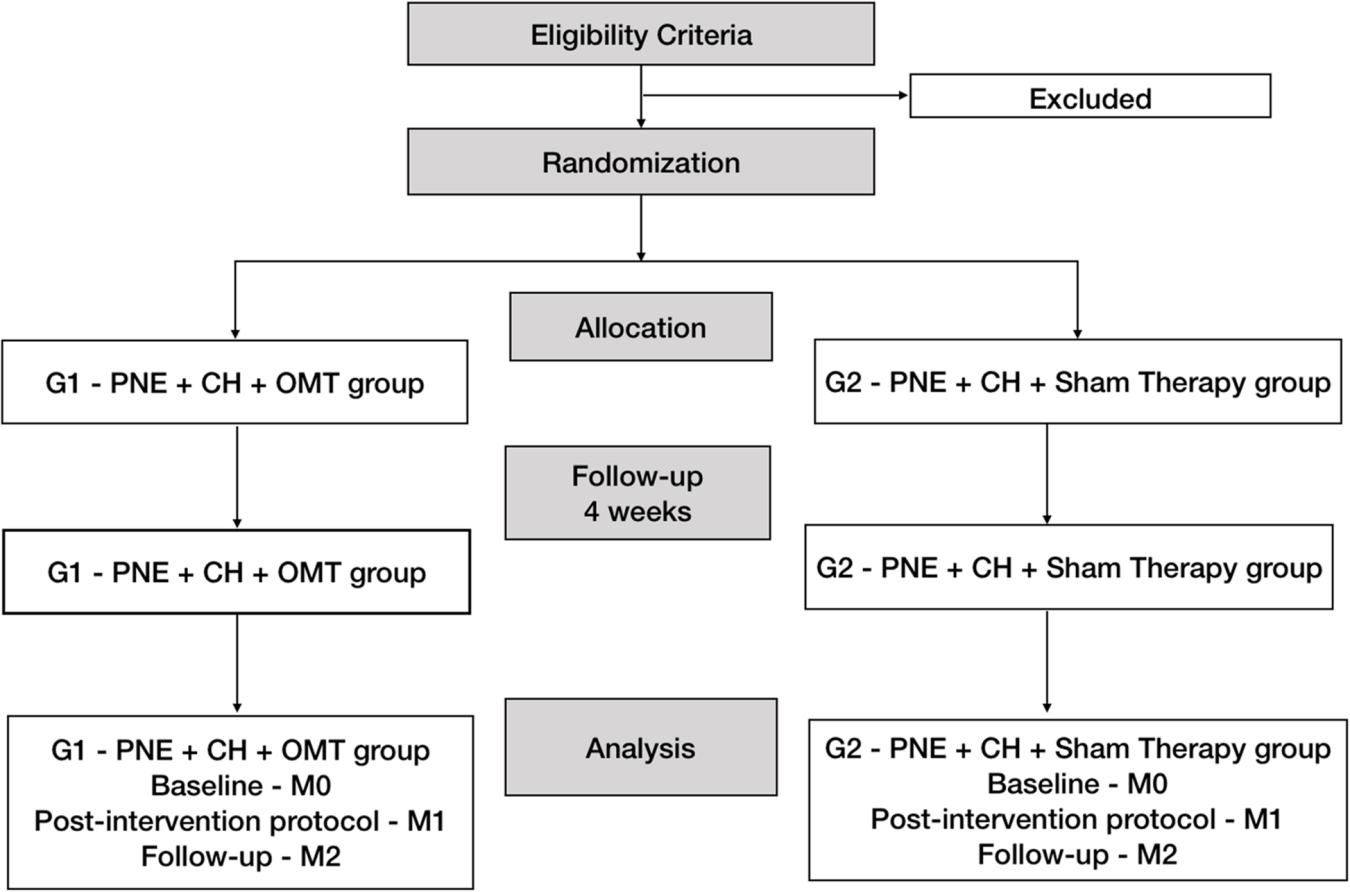
Study flowchart. PNE, pain education and neurosciences; CH, clinical hypnosis; OMT, osteopathic manipulative treatment; G1, intervention group; G2, control group

**Fig. 2:**
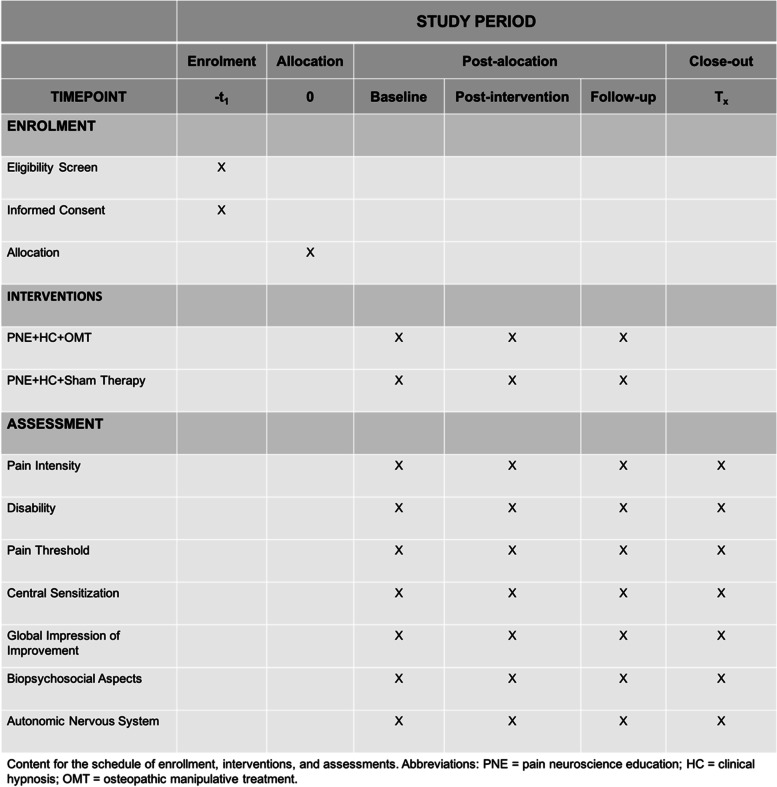
Schedule for enrollment, interventions and assessments

Participants will be randomly divided into two groups (Fig. 1 and Fig. 2), called PNE + CH + OMT (intervention group) and PNE + CH + sham therapy (control group). For both groups, the participants will be submitted to PNE based on information from the book “Explain Pain” and CH [11, 15]. The intervention group will receive the same approach and additionally four OMT visits and the control group, sham therapy. For both groups, the meetings of OMT or sham therapy will be distributed in 30 min and 20 final minutes with a focus on education with PNE and CH. I All procedures (control and intervention group) will be carried out individually, once a week, with an interval of at least 7 days. Participants who cannot attend on the pre-scheduled days will be offered new dates within the study period, in order to avoid sample loss. All participants will be encouraged to report adverse events at any time to any researcher during the conduction of this study.

### Pain neuroscience education (PNE)

Participants will receive four 20-min sessions, with an interval of 7 days between meetings, where the researcher will address PNE using metaphors, stories, and clear explanations about the neurophysiology of pain. Subsequently, the patient will be submitted to brief hypnotic experiences on the subjects taught in the PNE for greater fixation. At the end of the first consultation, participants will receive a 42-page printed booklet containing information covered in each meeting based on the book “Explain Pain” [15] and on their electronic devices, a 5-min video containing guided self-hypnosis performed during the session. Participants will be encouraged to read the information in the booklet and to practice guided self-hypnosis at home daily, between meetings [11]. The topics addressed at each meeting are described below.

- *First consultation*: The PNE theme to be addressed will be “Pain is a normal experience.” At the end of this explanation, participants will be subjected to hypnotic experiences to suggest openings for change, pointing out that the brain has many capacities that can be used to create comfortable experiences. Brief suggestions for exploring the capacities for relaxation, dissociation (body and mind), analgesia, regression, and previous pleasurable experiences will be addressed at this meeting.
- *Second consultation*: “Alarm system, its components and functions, in addition to the modulation of messages at the column level” will be the theme of the PNE at this meeting. In addition, two hypnotic experiments will be carried out, the first idea being expressed in the book Explain Pain - “Sensors are often replaced by new ones,” whose objective is to offer hypnotic experiences so that this adaptation in the sensors is possible. The second hypnotic experience will be related to the descending inhibitory modulation content at the spinal cord level, suggesting adapted hypnotic analgesia, inviting participants to experience analgesic sensations in their body.
- *Third consultation*: The PNE theme will correspond to “Changes in the central alarm system and the systemic pain response.” Next, two hypnotic experiments will be performed, the first linked to the idea that “the brain can receive amplified messages from the tissues, even when there is nothing wrong with the structure of the body,” including suggestions to lessen the discomfort of pain by inviting the patient to experience situations of comfort even in the face of some sensations in the body. The second experience will relate to the idea “the brain orchestra can play a different song.” We will offer sensory substitution suggestions, which invite participants to experience a comfortable sensation of their hands spreading to other parts of the body.
- *Fourth consultation*: The theme of this last PNE meeting will be “Understanding that injury is not the same as pain and education about gradual exposure.” In addition, in this last meeting, three hypnotic experiences will be given. The first will be a deep relaxation suggestion, in which participants will be asked to relax every muscle in their body. The second will be an “age regression and progression” experience, in which participants will be invited to bring adaptive responses to past or future pain into the present. Both experiences reinforce the importance of physical movement as a way to increase the benefits. The last hypnotic experience will offer a metaphor to help participants deal with negative thoughts throughout the day.

### Clinical hypnosis (CH)

The procedures for the hypnotic experiences performed right after the PNE will be divided into two parts, being the first the access to the state of consciousness that will facilitate the opening for changes. In this induction state, the researcher will invite the participant to focus their attention on a specific experience which may be the researcher’s voice or the participant's own breathing. In this state of focused attention and inhibition of critical thinking, they will be advanced to the second phase, which are suggestions for change. At that moment, specific therapeutic changes will be suggested, such as changes in sensations, behaviors, and thoughts that influence the perception of pain, based on the theme of each protocol consultation [16] previously described.

### Osteopathic manipulative treatment (OMT)

The researcher will perform a standard diagnostic palpatory assessment in order to identify somatic dysfunctions. Somatic dysfunctions are characterized by the functional alteration of the structures of the somatic system, which involve bone, joint, myofascial, vascular, lymphatic, and neural structures. The diagnostic criteria in the evaluation of somatic dysfunctions are expressed in the search (assessment) of asymmetries, changes in range of motion, and abnormalities in the textures of the tissues of the musculoskeletal system. The asymmetries will be evaluated looking for the height of the iliac crests, verification of the curvature of the lumbar spine, and rotation of the femorotibial axes through observation (static inspection) and palpation. Changes in range of motion will be assessed in joints, a group of joints or a region of the system through active and/or passive movement tests. Increased mobility (hypermobility) and decreased mobility (hypomobility) can be found. Abnormalities in tissue texture will be identified by palpatory procedures [17].

Based on the findings of the clinical evaluation, techniques addressing the musculoskeletal systems will be performed at each meeting [18] and/or visceral with direct anatomical correlation with the lumbar [18]. In the musculoskeletal system, the lumbar segment, pelvis, sacrum, and lower limbs will be addressed; these structures are directly connected by the lumbosacral plexus. The techniques used in these segments will be myofascial techniques and joint techniques. Myofascial techniques are techniques directed at the fascia or muscle and through approaches that use continuous palpatory feedback in order to assess and alleviate the somatic dysfunctions involved. Articulation techniques are direct manual techniques that impose movements that can be of low or high speed and low or moderate amplitudes in order to improve joint function. Visceral manipulation will be used in abdominal areas that might be in relationship with the ganglia of the lumbar area, namely renal aortic ganglion (kidneys and gonads), superior mesenteric ganglion (duodenum, jejunum, ileum, cecum, appendix, ascending colon and half of the transverse colon), and inferior mesenteric ganglion (distal part of the transverse colon, descending colon and rectum) [19]. This approach is characterized by manual techniques for diagnosing and treating the viscera and their supporting structures to improve their physiological function [19]. All manual approaches delivered will be recorded in the case report file (CRF).

It is noteworthy that the OMT will be applied only to participants randomly allocated to the intervention group, that is, they will receive OMT in addition to the PNE + CH in the four meetings.

### Sham therapy

The sham therapy will last 30 min. In the first 10 min, the researcher will perform the same evaluation described at OMT section, and in the remaining 20 min, the simulated therapy will be performed. The diagnostic criteria will follow the same steps of the evaluation of somatic dysfunctions, which will be expressed by the assessment of asymmetries, changes in range of motion, and abnormalities in the textures of the tissues of the systems. For the simulated therapy, the researcher will assess the anatomic areas, including the lumbar segment, pelvis, sacrum, and lower limbs, and for simulating the visceral treatment, the researcher will contact the abdominal pelvic quadrants, with superficial touch, without therapeutic intention [20, 21]. For each area, the researcher will mentally count from 120 to 0 to prevent placebo autonomic activation [22]. It is noteworthy that the sham therapy will be applied only to participants randomly allocated to the control group.

### Compliance participation

Participants will be encouraged to bring doubts about the information provided in the previous consultation, and at the beginning of each one, the possibility of doubts regarding the education in neuroscience of pain discussed above will be open. In addition, a review of the topics already discussed will be carried out. Also at that moment, the participants will be asked about the practice of self-hypnosis at home and the other orientations carried out. In this way, we aim to guarantee the participation and adherence of the volunteers in the proposed procedures.

Participants will be asked about the need for co-interventions, such as the use of analgesics or other physical therapies, during the intervals between consultations. All information must be informed to the researchers.

### Multilateral contributions to the development of the protocol

The development of the experimental protocol considered reports and clinical experiences of osteopaths, feedback, and opinions from patients, in addition to a literature review showing the multidimensional approaches.

### Outcomes

Participants will be evaluated by a researcher blind to allocation and the evaluative moments for all outcomes will correspond to the pre-intervention (M0), immediately after the last meeting (M1) and reassessed again in the follow-up of four (M2) weeks after the end of the protocols (Fig. 2).

#### Main outcome

Pain and disability will be the main outcomes of the study. Pain will be evaluated through the numerical pain scale, where the participant will be asked about their perception of pain suffered in the last week and will assign a value to it, ranging from 0 to 10, where 0 indicates no pain and 10 indicates worst pain ever felt [11, 23]. Pain will also be assessed by pressure threshold using a pressure algometer device (Fnd-50, PIAB 50-n, Italy) in the lumbar region. The assessment will be conducted specifically on the paraspinal muscles bilaterally at the L1 to L5 levels. Three measurements will be performed at the same point, and the average of the values will be calculated [24]. The distal end of the device will be positioned perpendicularly on the evaluated surface, and constant pressure of 2.55 kg will be applied. Participants will be instructed to indicate the moment when the sensation of pressure changes to a sensation of pain and the value will be recorded. The pain threshold will be defined in kilogram-force (kgf/cm^2^), through the minimum pressure necessary to induce pain in the evaluated region. Still as the main outcome, disability will be evaluated through the Oswestry Disability Questionnaire translated and validated for the Brazilian population [25] composed of ten questions that assess pain intensity and the effect of pain on activities of daily living and classify the individual as minimal (0–20%), moderate (21–40%), severe (41–60%), disability (61–80%), or bed-bound (81–100%) [26].

#### Secondary outcomes

As secondary outcomes, the patient’s global impression of improvement, central sensitization, and biopsychosocial factors will be evaluated. The patient’s overall impression of improvement will be assessed using the 11-point Improvement Percentage Scale, ranging from − 5, indicating “much worse,” to 0 indicating “no change” to +5 “completely recovered” [23, 27], i.e., higher scores correspond to greater recovery. Central sensitization will be evaluated using the Central Sensitization Questionnaire translated and validated for the Brazilian population [28] composed of two parts (A and B), where part A contains 25 statements, scored on a Likert scale from 0 to 4 points, whose final score can vary from 0 t o100 points, with values closer to 100 indicating a greater degree of central awareness. Part B contains a list of 10 medical diagnoses related to central sensitization syndrome, and the subject is asked about the presence/absence and year of diagnosis. Biopsychosocial factors will be evaluated using the STarT Back Toll questionnaire [29] which classifies the risk of poor prognosis of individuals with LBP in the presence of physical and psychosocial factors and is composed of nine items related to referred pain, dysfunction, comorbidities, and psychosocial factors such as catastrophizing, fear, anxiety, and depression that classify the individual as low, medium, and high risk.

In addition, the autonomic nervous system will be evaluated through the heart rate variability (HRV), which will be analyzed using linear methods, in the domains of time (SDNN and RMSSD) and frequency ((LF ms2, LF un, HF ms 2 , HF un and LF/HF) and by the geometric indices (SD1, SD2 and SD1/SD2) [30]. The participant will be invited to lie in the supine position on a stretcher, and the evaluator will place a heart rate monitor (Polar RS800CX) on the individual’s chest, with the objective of recording the heart rate beat by beat for 15 min, for later analysis of the HRV. After capturing the heart rate beat by beat, by the Polar RS800CX heart rate monitor (Polar Electro, Kempele, Finland), the data will be submitted to digital (Polar Pro Trainer software version 5.0) and manual filtering (Microsoft Office Excel version 15.3) to eliminate premature ectopic beats and artifacts [31], and only sets with more than 95% of sinus beats will be included in the study. From the period of greater stability of the tachogram, 1000 consecutive RR intervals will be extracted from the baseline and final moments, followed by the calculation of HRV indices using the Kubius HRV software [32]. For the spectral analysis (LF ms2, LF u.n, HF ms 2, HF u.n and LF/HF), the fast Fourier transform algorithm will be used [33].

### Participant timeline

The flowchart of the time schedule, interventions, assessments, and visits for participants is shown in Fig. 2.

### Sample size

The sample size was calculated to detect an effect between groups for the pain intensity outcome assessed using the numerical scale. For the calculation, a power of 80% and an alpha error of 5% were adopted and, based on the results obtained by Rizzo et al. [11], a standard deviation of 2.0 points and an average difference of 1.3 points were considered, which resulted in a total sample size of 52 individuals. Considering possible sample losses, there will be an increase of 15% in the sample size calculated, totalizing 60 participants. All calculations were performed on the software G*Power version 3.1.9.7 [34].

### Recruitment

Participants with CLBP will be recruited from the waiting list of the clinical physiotherapy outpatient clinic at the State University of the North of Paraná, in the city of Jacarezinho, Paraná, Brazil, and through social media advertisements.

### Allocation—sequence generation

A simple randomized sequence will be created via the www.randomization.com website by a contributor external to the researchers directly involved in the research. After the initial assessment, the researcher will access the randomization envelope and carry out the intervention according to the group allocation indicated on the envelope, that is, group 1 PNE+CH+OMT or G2 PNE + CH + sham therapy.

### Allocation concealment mechanism

The allocation will be concealed using opaque, sealed, numbered envelopes by a contributor external to the researchers directly involved in the research.

### Blinding

The person responsible for evaluating the outcome measures will be blinded to the allocation of intervention groups. Given the nature of the study, it is impossible to blind the researcher/therapist. At the end of the protocol, participants will be asked to report on which type of manual therapy was assigned to their treatment (sham or OMT), in order to assess blinding success [35].

### Statistical analysis

To analyze the population profile data, we will use the descriptive statistical method and the results will be presented with mean values, medians, standard deviations, minimum and maximum values, and confidence intervals.

Data normality will be determined using the Shapiro-Wilk test, and the comparison of variables between groups at baseline will be performed using Student’s *t*-test for unpaired data for data with normal distribution or the Mann-Whitney test for non-normal data.

Comparisons of primary and secondary outcomes between groups and moments will be performed using the analysis of variance technique for repeated measures model in the two-factor scheme. Repeat measurement data will be checked for sphericity violation using Mauchly’s test, and the Greenhouse-Geisser correction will be used when sphericity is violated.

To analyze the moments, Bonferroni’s post-test for normal distribution or Dunnet’s post-test for non-normal distribution will be used, and the analysis of the different moments between the groups will be carried out using Student’s *t*-test for unpaired data with normal distribution or Mann-Whitney test for non-normal data.

The adopted statistical significance will be set at 5%. Analyses will be performed using the Statistical Package for the Social Sciences software—version 22.0 (SPSS Inc., Chicago, IL, USA) by a researcher blinded to treatments and group allocations using a coded spreadsheet. All the randomized participants will be included in the analysis, in accordance with intention-to-treat principles. To consider all participants, the missing data will be addressed according to the last observation carried forward (M0 or M1) [36]. Analysis and results from this study will be conducted by an independent biostatistician and checked for accuracy.

### Auditing

The Research Ethics Committee of the State University of the North of Paraná (UENP) will check during the project the presence and the completeness of the investigation files, such as inclusion and exclusion criteria, informed consents, data collections, and storage.

### Protocol amendments

All substantial amendments will be notified to the ethics committee of the State University of the North of Paraná (UENP). If amendments concern or affect participants, they will be informed about the changes, and if needed, additional consent will be requested and registered. Also, online trial registries will be updated accordingly.

### Confidentiality

All data extracted from the participants are strictly confidential, being related to the participants through codes, to which only the researchers will have access, guaranteeing their confidentiality. The data extracted from the participants regarding the characterization and outcomes of this study will be recorded in individual forms and later stored in digital spreadsheets (Microsoft Office), ensuring the same secrecy and confidentiality mentioned above.

### Data access

The access to the final trial dataset will be available in a repository from the State University of the North of Paraná (UENP).

### Ancillary and post-trial care

The clinic of the State University of the North of Paraná will be available to all participants of this study for follow-ups of their clinical status. In addition, all participants who wish to continue treatment, sham therapy or OMT, will be placed on the clinic’s registration list.

### Dissemination plan

The researchers consider data sharing to be an important component of clinical research. All the data will be published in international peer-reviewed journals. In addition, the results of this study will be communicated to the participants, healthcare professionals, and on social media. We also plan to present our data at relevant international congresses and conferences across South America and, if possible, in other continents.

### Implementation plan

From the results obtained with the execution of the protocol, it is intended to consider the elaboration of strategies on the importance and implementation of the main themes of the study, in the treatment of the patients of the clinic of the State University of the North of Paraná.

## Discussion

### Potential impacts and significance of the study

The study will provide information on the effectiveness of osteopathic manipulation combined with education in pain neuroscience and clinical hypnosis on pain parameters, disabilities, and biopsychosocial aspects of patients with DLC, verifying the importance or not of including “hands on” approaches.

The guidelines have been encouraging multimodal, biopsychosocial approaches for patients with CLBP; in this sense, the results of this study can help clinicians and researchers in the implementation of a model of treatment strategy for these patients. If the study hypothesis is proven, patients may benefit from approaches with minimal risk of deleterious effects and low cost. In addition, it will enable the addition of relevant elements to the literature, with approaches that interact and do not segment the body and brain of patients with CLBP, allowing new studies in this scenario.

### Contribution and clinical applicability

The study design discusses a model of manual therapy, OMT, which proposes a standard diagnostic assessment and treatment based on these clinical findings. This type of therapy aims at a cascade of ascending neurophysiological effects (bottom-up) that reach the higher centers, helping to control symptoms. Added to this, the biomechanical effects and potential placebo effects stand out in this type of approach. Thus, the “bio” of biopsychosocial approaches to patients with CLBP is considered.

Together, clinicians, in addition to modulating ascending information, can use PNE to generate descending effects, that is, from the brain to the tissues (top-down). It is known through pain neuroscience studies that patients with chronic pain have structural and functional changes in the brain. And the educational processes have been proving to be effective for patients in the knowledge of their condition and improving the psychological and behavioral aspects in relation to pain.

The protocol is supplemented with clinical hypnosis and self-hypnosis practices. With this therapy, the objective is to favor change processes, expand sensations and behaviors, break limiting beliefs, and learn and change through access to the unconscious. Clinical hypnosis can be a self-applied tool to manage pain-related anxiety and depression, enhancing the effects of pain education. With these practices, the “psychosocial” of biopsychosocial practices is respected.

For clinicians, these findings may provide information about the possibility of using multimodal approaches in the management of patients with CLBP. For patients and the general population, it can demonstrate the benefits of manipulations and active movements and provide knowledge (education in pain neuroscience) and consequent active participation in the rehabilitation process and demystification of hypnosis processes. Finally, the proposal is an approach that can reduce economic costs for patients and put less burden on health insurance and the state.

### Trial status

Protocol number: NCT05042115.

The recruitment process is ongoing; it started in November 2021, and we intend to finish it in May 2022.

## Abbreviations

CLBP: Chronic low back pain
OMT: Osteopathic manipulative treatment
PNE: Pain neuroscience education
CH: Clinical hypnosis
LBP: Low back pain
SPIRIT: Standard Protocol Items: Recommendations for Interventional Trials
NPE: Numerical pain scale
ICF: Informed consent form
CRF: Case report file
M0: Pre-intervention
M1: Immediately after the last meeting
M2: Reassessed again in the follow-up of four weeks after the end of the protocols
HRV: Heart rate variability
